# 27-gauge trocar-assisted sutureless intraocular lens fixation

**DOI:** 10.1186/s12886-020-01758-6

**Published:** 2021-01-06

**Authors:** Tatsuya Jujo, Jiro Kogo, Hiroki Sasaki, Reio Sekine, Keiji Sato, Sakura Ebisutani, Yasuhiro Toyoda, Yasushi Kitaoka, Hitoshi Takagi

**Affiliations:** Department of Ophthalmology, St. Marianna University School of Medicine, 2-16-1 Sugao, Miyamae-ku, Kawasaki, Kanagawa Japan

**Keywords:** Sutureless intraocular lens fixation, 27-gauge trocar, Flange technique, 27G vitrectomy, CASIA2

## Abstract

**Backgrounds:**

However there have been numerous investigations of intrascleral intraocular lens (IOL) fixation techniques, there is room for improvement in terms of simplifying complicated techniques and reducing the high levels of skill required. This study aimed to report a novel technique for sutureless intrascleral fixation of the IOL using retinal forceps with a 27-gauge trocar.

**Methods:**

Nineteen eyes of 18 patients underwent intrascleral fixation of the IOL from July 2018 to September 2019 were enrolled in this study. A 27-gauge trocar formed 3-mm scleral tunnels positioned at 4 and 10 o’clock, 2 mm from the corneal limbus. We used a 3-piece IOL haptic grasped by a 27-gauge retinal forceps and pulled from the 27-gauge trocar. The IOL was fixed by making a flange. Main outcome measures were visual acuity, corneal endothelial cell density, IOL tilt, decentration, predicted error of refraction and complications.

**Results:**

The 19 eyes were followed up for 1 month. The mean pre- and postoperative logMAR uncorrected visual acuity (UCVA) was 1.06 ± 0.63 and 0.40 ± 0.26, respectively (*p* < 0.01), while the mean pre- and postoperative logMAR best corrected visual acuity (BCVA) was 0.27 ± 0.51 and 0.06 ± 0.15, respectively (*p* = 0.09). The mean corneal endothelial cell density was 2406 ± 625 to 2004 ± 759 cells/mm^2^ at 1 month (*p* = 0.13). The mean IOL tilt was 3.52 ± 3.00°, and the mean IOL decentration was 0.39 ± 0.39 mm. There was no correlation among IOL tilt, decentration and BCVA (*p* > 0.05). The mean prediction error of the target refraction was − 0.03 ± 0.93 D. The complications were vitreous hemorrhage (3 eyes), hyphema (1 eye), IOP elevation (1 eye), iris capture of the IOL (1 eye) and hypotony (2 eyes). No IOL dislocation occurred.

**Conclusions:**

IOL intrascleral fixation with a flange achieved good IOL fixation and visual outcome in the scleral tunnels created with the 27-gauge trocar.

## Background

The pseudophakic population has been growing very rapidly in recent years as a result of longer lifespans, new phacorefractive procedures and improvements in the quality and safety of phacoemulsification surgery [1]. Intraocular lens (IOL) dislocation is a late complication after cataract surgery. It was reported that 20 years after cataract surgery, 10 of 800 patients at risk (1.2%) needed dislocation surgery [2]. As a result, cases of IOL dislocation are expected to increase annually.

IOL dislocation causes a variety of complications. For example, increased intraocular pressure, rhegmatogenous retinal detachment, and vitreous hemorrhage may occur and urgent surgery is required [3, 4]. There are two procedures to correct IOL dislocation. One is the use of ab externo suture techniques to guide the sutures through the sclera to fix the IOL, as first reported by Malbran et al. in 1986 [5]. The other is the sutureless intrascleral fixation of posterior chamber-IOL implantation. IOL haptics are embedded in prepared scleral tunnels, thus stabilizing the posterior chamber-IOL [6]. While the long-term outcomes of intrascleral IOL fixation are unsatisfactory, the intrascleral IOL fixation technique results in less IOL tilt and decentration and a better iris capture rate than external-suture IOL fixation techniques [7].

There have been numerous investigations of intrascleral IOL fixation techniques [6–14]. Totan and Karadag improved previous on techniques by making scleral tunnels with the insertion of 25-gauge transconjunctival sutureless vitrectomy microcannulas using trocars and then closing the tunnels with a 10–0 monofilament transconjunctival suture [14]. Recently, the double-needle technique has been used to form scleral tunnels without sutures by creating a flange [7].

We developed a novel sutureless scleral IOL fixation technique using a 27-gauge trocar with a flange. This technique does not require scleral tunnel sutures and is easy to perform using double forceps with the 27-gauge trocar. Here we report the 1-month follow-up results in 19 eyes that underwent this procedure.

## Methods

### Patients

This retrospective study was approved by the Institutional Review Board of St. Marianna University School of Medicine and adhered to the tenets of the Declaration of Helsinki. Written informed consent to participate in the current study was obtained from all patients. We registered this study in University hospital Medical Information Network (https://www.umin.ac.jp) (UMIN 000040437).

Nineteen eyes from 18 patients with aphakia, a dislocated IOL or subluxated crystalline lens were included in this retrospective study. The specific diagnoses were IOL dislocation/fall in 12, subluxated crystalline lens in 3 and aphakia in 4 eyes. The study exclusion criteria were hazy cornea or corneal scarring, aniridia, macular scarring, glaucoma, traumatic history and postoperative follow-up for less than 1 month. All surgeries were performed at St. Marianna University of Medicine between July 2018 and September 2019.

### Surgical technique

**Fig. 1:**
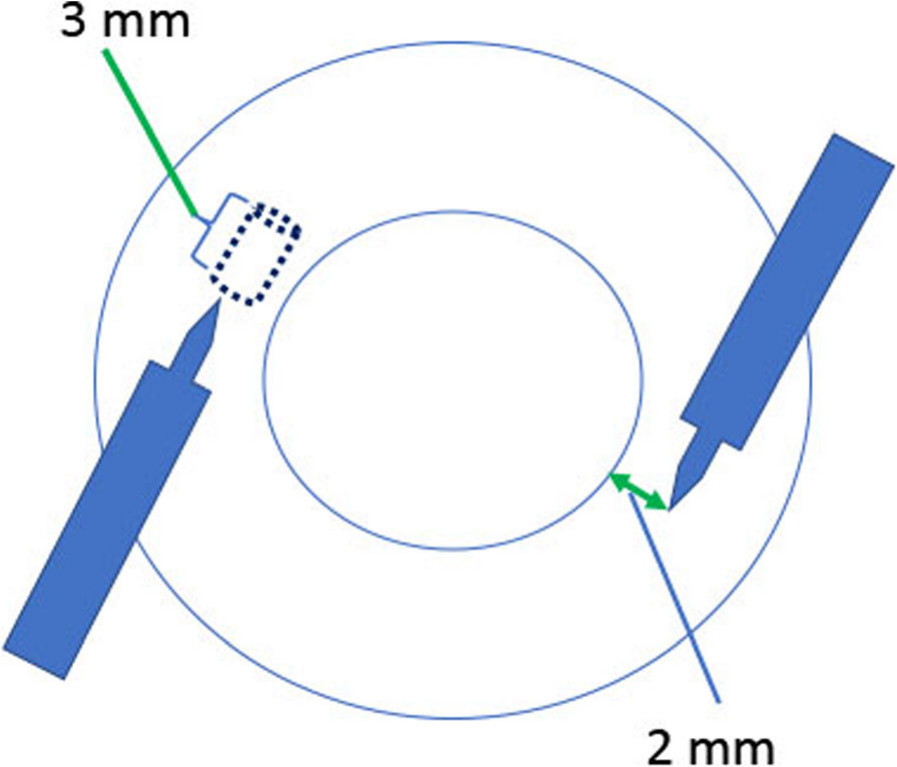
Scleral tunnels were formed with a 27-gauge trocar inserted 2 mm from the corneal limbus at a 10° angle. The parallel scleral tunnels at the 4 o’clock and 10 o’clock positions were 3 mm in length

**Fig. 2:**
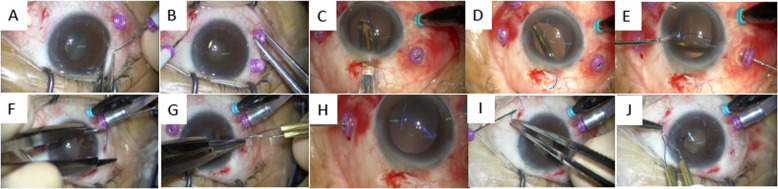
27-Gauge trocar-assisted sutureless intraocular lens fixation procedure. All procedures were performed by the 27-gauge microincision vitrectomy system including a three-port trocar cannula system. Infusion cannula was used for all cases. **a**, **b**: The 27-gauge trocar was used to form 3-mm scleral tunnels 2 mm from the corneal limbus at the 4 o’clock and 10 o’clock positions. **c**: The IOL was inserted into the anterior chamber using an injector. **d**: The trailing haptic was held outside to prevent the IOL from falling into the vitreous cavity. **e**: A 23-gauge sideport forceps was used to grasp the trailing haptic, and then 27-gauge retinal forceps were inserted into the anterior chamber through the 27-gauge trocar to grasp the haptic end. **f**: The 27-gauge trocar was first pulled back, and then the 27-gauge retinal forceps grasped and pulled the trailing haptic through the scleral tunnel. **g**: The haptic end was used to form a flange with an ophthalmic cautery device. The flange was then pushed into the scleral tunnel. (**h**, **i**, **j**): The same procedures as in **e**, **f** and **g** were performed on the opposite side

All procedures were performed using the 27-gauge microincision vitrectomy system using the Alcon Constellation Vision System (Alcon Laboratories, Inc., Fort Worth, TX, USA) including a three-port trocar cannula system (Total Plus Pak). Infusion cannula was used for all cases. The surgical procedure is summarized as follows: 1) Scleral tunnels were made with a 27-gauge trocar 2 mm from the corneal limbus at the 4 o’clock and 10 o’clock positions. The tunnel length was 3 mm transconjunctivally at a 10° angle (Fig. 1, Fig. 2 a.b). 2) A 3-piece IOL (NX-70, Santen, Osaka, Japan) was inserted into the anterior chamber using an injector, and the trailing haptic was held outside to prevent the IOL from falling into the vitreous cavity (Fig. 2 c.d). 3) A 27-gauge retinal forceps was inserted into the posterior chamber through a prepared 27-gauge cannula, and a 23-gauge sideport forceps was inserted into the anterior chamber through the paracentesis to grasp the trailing haptic. The haptic was then moved toward the 27-gauge retinal forceps until it could be grasped and the trailing end was pulled through the scleral tunnel (Fig. 2 e.f.h.i). 4) The end of the haptic was formed into a flange using an ophthalmic cautery device (Accu-Temp Cautery; Beaver Visitec, Waltham, MA, USA). The flange was then inserted into the scleral tunnel (Fig. 2 g.j).

### Examination

**Fig. 3:**
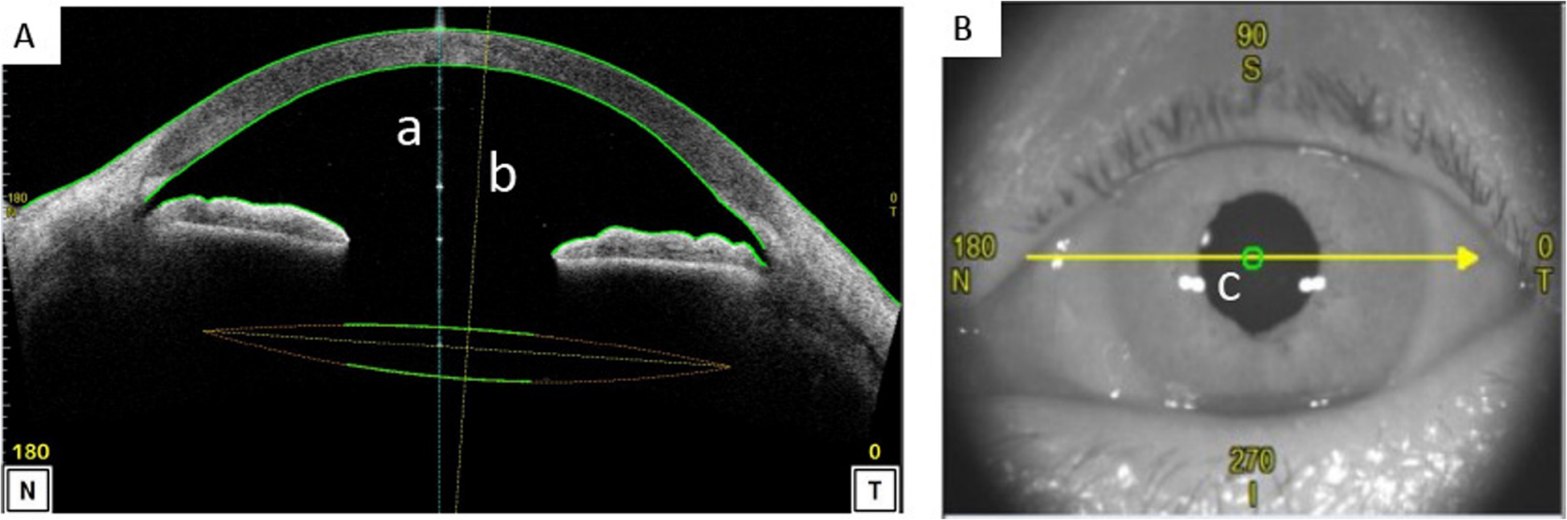
Measurement of tilt and decentration. The tilt and decentration of the IOL were quantified using a second-generation anterior segment optical coherence tomography (AS-OCT) system. A, Sagittal image of the cornea taken with the AS-OCT system. B, Charge-coupled device image taken with the AS-OCT system. The tilt was defined as the angle between the corneal topographic axis and central axis of the IOL. The corneal topographic axis is a reference line that connects the fixation point on the corneal topographer to the corneal vertex (**a**). The central axis of the IOL is a vertical line from the center of the IOL (**b**). Similarly, decentration was defined as the distance between the fixation point on the corneal topographic axis and central axis of the IOL in the cornea (**c**)

Preoperatively and 1 month postoperatively, all patients underwent an ophthalmological examination including the logarithm of minimal angle of resolution (logMAR) uncorrected visual acuity (UCVA), logMAR best corrected visual acuity (BCVA), slit-lump biomicroscopy, IOP measurement, manifest refraction and corneal endothelial cell density. All IOL powers were calculated by the SRK-T formula with a standardized A constant using an IOL Master (Carl Zeiss Meditec, Jena, Germany). The tilt and decentration of the IOL were quantified using a second-generation anterior segment-optical coherence tomography (OCT) system (Casia2, Tomey Corporation, Nagoya, Japan). The tilt was defined as the angle between the corneal topographic axis and central axis of the IOL. Similarly, decentration was defined as the distance between the corneal topographic axis and central axis of the IOL (Fig. 3). IOL tilt was calculated in the axial direction of 158–338°, which was the approximate position of the scleral tunnels.

### Statistical analysis

Data were analyzed using IBM SPSS Statistics version 21.0 (IBM Corp., Armonk, NY, USA). The Wilcoxon signed-rank test was used for comparisons of logMAR UCVA and BCVA, and corneal endothelial cell density was compared between the preoperative and postoperative values. The correlations between IOL tilt and decentration, logMAR BCVA and IOL tilt, and logMAR BCVA and decentration were analyzed using Pearson’s test. *p* values of less than 0.05 were considered to represent statistically significant differences.

## Results

The patient characteristics are shown in Table 1. Nineteen eyes of 18 patients underwent this surgical procedure. The mean patient age was 71.2 ± 14.4 years. Fifteen (79%) of the eyes were from men and 4 (21%) from women. The diagnosis was aphakia in 4 eyes (21%), dislocated posterior chamber IOL in 12 eyes (63%) and subluxated crystalline lens in 3 eyes (16%). None had macular disease or glaucoma.

**Table 1:** Patient characteristics

Characteristics		Mean (± SD)
No. of eyes		19
Age (years)		71.2(±14.4)
Gender (male/female)		15/4
Diagnosis (no. of eyes)	・Aphakia	4
	・Dislocated PC IOL	12
	・Subluxated crystalline lens	3
Axial length (mm)		24.65 (± 1.91)
Preoperative BCVA (logMAR)		0.27 (± 0.51)

*SD* Standard deviation; *logMAR* Logarithm of minimum of resolution; *BCVA* Best corrected visual acuity; *PC IOL* Posterior chamber intraocular lens

**Fig. 4:**
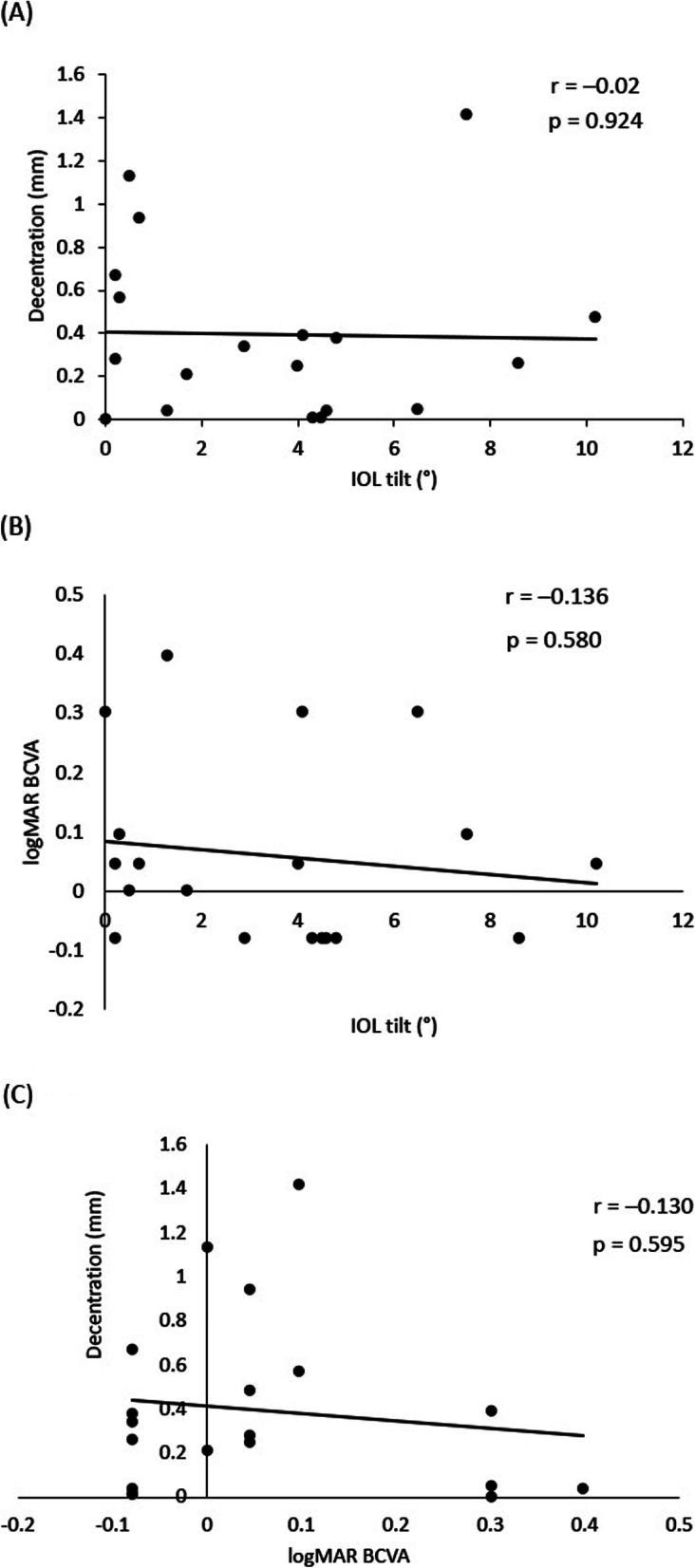
Correlation between visual acuity, tilt and decentration. Pearson’s test was used to determine the correlation between tilt and decentration (**a**), tilt and best corrected visual acuity (BCVA) (**b**) and BCVA and decentration (**c**). The correlation coefficient between tilt and decentration was − 0.02, that between tilt and BCVA was − 0.136 and that between BCVA and decentration was − 0.130. No correlation was observed among visual acuity, tilt and decentration

Table 2 shows the surgical outcomes in terms of visual acuity, corneal endothelial density, IOL tilt and decentration. Postoperative logMAR UCVA (0.40 ± 0.26) improved significantly compared with preoperatively (*p* < 0.01). The mean pre- and postoperative logMAR BCVA was 0.27 ± 0.51 and 0.06 ± 0.15, respectively (*p* = 0.09). The mean corneal endothelial cell density was 2406 ± 625 to 2004 ± 759 cells/mm^2^ at 1 month postoperatively (*p* = 0.13). The mean IOL tilt was 3.52 ± 3.00°, and the mean IOL decentration was 0.39 ± 0.39 mm. The correlations between IOL tilt and decentration (*r* = 0.020, *p* = 0.924) (Fig. 4a), logMAR BCVA and tilt (*r* = − 0.136, *p* = 0.580) (Fig. 4b) and logMAR BCVA and decentration (*r* = − 0.130, *p* = 0.595) (Fig. 4c) were not significant.

**Table 2:** Surgical outcomes

	Preoperative (mean ± SD)	Postoperative (at 1 month) (mean ± SD)	*p* value
logMAR UCVA	1.06 ± 0.63	0.40 ± 0.26	< 0.01
logMAR BCVA	0.27 ± 0.51	0.06 ± 0.15	0.09
Corneal endothelial cell density (cells/mm^2^)	2406 ± 625	2004 ± 759	0.13
Tilt (°)		3.52 ± 3.00	
Decentration (mm)		0.39 ± 0.39

Table 3 shows the prediction errors of the postoperative refraction compared with the IOL Master target refraction. The mean prediction error of the target refraction was − 0.03 ± 0.93 D.

**Table 3:** Prediction error of target refraction

*n* = 19	Mean ± SD	Median (minimum–maximum)
Prediction error (D)	−0.03 ± 0.93	−0.02 (− 1.28 to 1.62)

The most frequent postoperative early complication was vitreous hemorrhage in 3 eyes (16.8%). This resolved within 1 week in 2 eyes, but 1 eye required surgical intervention to treat prolonged bleeding and IOP elevation. Other complications were hyphema in 1 eye (5.3%), IOP elevation (> 25 mmHg) in 1 (5.3%), iris capture of IOL in 1 (5.3%) and hypotony (< 5 mmHg) in 2 (10.5%) (Table 4). These recovered without medication within 1 week. IOL dislocation and other complications were not observed during the follow-up period.

**Table 4:** Postoperative early complications

Complication	No. of eyes (%), *n* = 19
Vitreous hemorrhage	3 (16.8)^a^
Hyphema	1 (5.3)
IOP elevation	1 (5.3)
Iris capture of IOL	1 (5.3)
Hypotony	2 (10.5)
CME	0
Dislocated IOL	0

*IOP* Intraocular pressure; *IOL* Intraocular lens; *CME* Cystoid macular edema

^a^1 eye required reoperation because bleeding was prolonged

## Discussion

Various intrascleral IOL fixation techniques have been developed in recent years. Although safety and good fixation of the IOL with these intrascleral fixation methods were reported, there is room for improvement in terms of simplifying complicated techniques and reducing the high levels of skill required. Totan and Karadag described a technique for making scleral tunnels prepared by insertion of 25-gauge transconjunctival sutureless vitrectomy microcannulas using trocars, and this technique provided good IOL stabilization with a shorter surgical time [14]. We believed that using 27-gauge trocars to make the scleral tunnels for intrascleral IOL fixation with the flange technique [7] might be safer and more easily performed due to the smaller surgical wound and direct approach to the IOL loop with the intraocular forceps. Similarly, David and Juan reported a sutureless IOL scleral fixation technique using a 27gauge trocar. This study reported good postoperative visual acuity over a 12-month observation period [15]. Here, we reported the clinical results in a series of more patients who underwent our novel intrascleral fixation method, as well as detailed IOL fixation results using anterior segment-OCT.

The mean preoperative logMAR UCVA in our patients improved 1 month postoperatively compared with baseline. This study was the first to measure intrascleral IOL fixation tilt and decentration with anterior segment-OCT. The tilt and decentration of the IOL were measured at the corneal topographic axis because the corneal vertex is not affected by the shape of the pupil and therefore that axis is a better reference for assessing tilt and decentration than the pupil center. In this study, the mean postoperative IOL tilt was 3.52 ± 3.00° and decentration was 0.39 ± 0.39 mm. Kimura et al. reported that the crystalline lens and the IOL showed an average tilt of 4–6° toward the inferotemporal direction relative to the corneal topographic axis and an average decentration of less than 0.12 mm [16]. Therefore, our technique resulted in greater IOL tilt and decentration than occurs in normal cataract surgery. We could not compare our results with other reports of intrascleral IOL fixation because the IOL tilt and decentration were not determined in a similar manner. There was no significant difference between the mean number of corneal endothelial cells before and after surgery, although a declining trend was noted in this study. It was reported that 1 month postoperatively, corneal endothelial cell loss was 10.1% in eyes that underwent planned extracapsular cataract extraction with posterior chamber IOL implantation [17]. Yamane et al. reported that the corneal endothelial cell density with the double-needle technique was 2341 ± 481 cells/mm^2^ preoperatively and 2313 ± 462 cells/mm^2^ at 6 months postoperatively, showing no significant decrease [7]. The difference between Yamane et al.’s [7] and our results could be related to the observation time point. In addition, the method used to grasp and pull out the IOL haptics directly might have affected the corneal endothelial cells, although no direct attachment occurred in our series of patients.

IOL tilt and decentration were not correlated with BCVA. Some groups reported that tilt and decentration after IOL implantation impaired visual quality and led to higher-order aberrations [18, 19]. However, it was also found that IOL decentration did not influence VA in eyes with monofocal IOLs [20] and that in-the-bag IOL that maintained a decentration of < 1 mm and an angle of < 4° did not influence BCVA [21]. The present study also suggested that IOL tilt and decentration did not affect visual acuity. However, the correlation could not be proved because the number of cases were small and the observation period was short.

The prediction error of the target refraction did not show a myopic shift trend and postoperative refraction was not stable in this study. After transscleral fixation of the IOL in aphakic vitrectomized eyes, a − 1.0 D myopic shift was seen 6 months postoperatively [13]. In the transscleral procedure reported, the IOL haptic is sutured 1.5 mm from the corneal limbus [13, 22], with scleral tunnels made 2 mm from the limbus, and thus no myopic shift occurred. The needle insertion angle is also important in fixing the IOL [7]. The unstable postoperative refraction seen in our patients was likely due to the gap that occurred between the insertion angle and insertion position when forming scleral tunnels.

Vitreous hemorrhage and hypotony occurred more frequently among our patients than in previous studies of transscleral IOL suture and intrascleral IOL fixation [7–13, 23]. The inner diameter of the 27-gauge trocar is 0.4 mm (27 gauge), but the outer diameter is 0.5 mm (25 gauge). Previous studies used 9–0 polypropylene and 27- or 30-gauge needles, and thus the cause of hemorrhage is believed to be related to the wound size. Our technique was based on the method using a 25-gauge transconjunctival sutureless vitrectomy trocar reported by Totan and Karadag [14]. However, our postoperative complications could not be compared because they did not report them. We also performed surgery by referring to the handshake technique, which Agarwal et al. originally reported, grasping the IOL haptic with forceps and pulling it out of the eye [23]. It is essential to hold the haptic at the tip so that it does not snag on the sclerotomy wound during externalization. Because we did not note this point in some cases, further study is necessary to assess it.

Rizzo et al. reported a study showing that 27-gauge sclerotomies resulted in good wound closure with a 1-step insertion at an angle of 30° [24]. Suturing of the sclerotomy site was unnecessary, and postoperative hypotony did not occur [24]. Mitsui et al. reported that after 27-gauge vitrectomy for epiretinal membrane, the scleral wounds closed at 7.7 ± 4.7 weeks [25]. Recently, we reported that the 27-gauge trocar wound closing occurred by postoperative day 10 [26]. Therefore, we thought that IOL fixation in the sclera was completed within 1 month in our study. Since hypotony was observed in a few patients, pulling out the IOL haptics with a trocar might affect wound closure even with a 27-gauge system. We need further detailed analysis of wound closure using OCT to assess this point.

We created a flange to prevent the haptics of the IOL from detaching. The outer diameter of the 27-gauge trocar is 0.5 mm, which ensures that the flange is large enough to fix the haptic securely [7]. All IOLs used in this study were the NX-70 type, and the flanges were made by heating the haptics. Scleral tunnels were formed at the 4 o’clock and 10 o’clock positions to prevent damage to the long ciliary nerves at the 3 o’clock and 9 o’clock positions [11].

Recently, Diamint and Giannbruni reported a similar surgical procedure. And they showed that the technique is safe, minimally invasive and relatively easy to perform [15]. Our procedure is a modification of the handshake technique, which involves grasping the IOL haptic with forceps and pulling it out of the eye [23]. An advantage of the handshake technique is that it does not require the insertion of a haptic, which is the most difficult element of the double-needle technique, into the needle. The handshake technique can also be applied in numerous clinical situations. For example, it is possible to cope with the IOL fall reported as a complication during intrascleral fixation, since the IOL that has fallen into the vitreous can be pulled out of eye by grasping and lifting the haptic using vitreous forceps. Also, in the case of the double-needle technique, the needle tip may reach the retina and ciliary body after the puncture, but with the present method, that risk is less because the forceps are manipulated under direct vision.

## Limitations

The limitations of this study were that the long-term postoperative course was not observed, and the number of cases was small, with only 19 eyes. Therefore, there is a possibility of unexpected postoperative complications. Moreover, there are unclear points about long-term IOL fixation due to the short-term observation. It will be necessary to continue to follow up the patients and to increase the number of cases.

## Conclusion

IOL intrascleral fixation with a flange achieved good fixation and visual outcomes when scleral tunnels were created with a 27-gauge trocar. However, postoperative vitreous hemorrhage and hypotony were seen more frequently in this small patient group than was reported in previous studies.

## Data Availability

The datasets used and/or analysed during the current study are available from the corresponding author on reasonable request.

## Abbreviations

IOL: Intraocular lens
IOP: Intraocular pressure
logMAR: Logarithm of minimal angle of resolution
UCVA: Uncorrected visual acuity
BCVA: Best corrected visual acuity
OCT: Optical coherence tomography
